# Artificial intelligence-based HDX (AI-HDX) prediction reveals fundamental characteristics to protein dynamics: Mechanisms on SARS-CoV-2 immune escape

**DOI:** 10.1016/j.isci.2023.106282

**Published:** 2023-02-27

**Authors:** Jiali Yu, Ugur Uzuner, Bin Long, Zachary Wang, Joshua S. Yuan, Susie Y. Dai

**Affiliations:** 1 Synthetic and Systems Biology Innovation Hub, Texas A&M University, College Station, TX 77843, USA; 2 Department of Plant Pathology and Microbiology, Texas A&M University, College Station, TX 77843, USA; 3 Department of Molecular Biology and Genetics, Karadeniz Technical University, Trabzon 61080, Turkey; 4 Department of Energy, Chemical and Environmental Engineering, Washington University at St Louis, St Louis, MO 63112, USA

**Keywords:** Immunology, Virology

## Abstract

Three-dimensional structure and dynamics are essential for protein function. Advancements in hydrogen-deuterium exchange (HDX) techniques enable probing protein dynamic information in physiologically relevant conditions. HDX-coupled mass spectrometry (HDX-MS) has been broadly applied in pharmaceutical industries. However, it is challenging to obtain dynamics information at the single amino acid resolution and time consuming to perform the experiments and process the data. Here, we demonstrate the first deep learning model, artificial intelligence-based HDX (AI-HDX), that predicts intrinsic protein dynamics based on the protein sequence. It uncovers the protein structural dynamics by combining deep learning, experimental HDX, sequence alignment, and protein structure prediction. AI-HDX can be broadly applied to drug discovery, protein engineering, and biomedical studies. As a demonstration, we elucidated receptor-binding domain structural dynamics as a potential mechanism of anti-severe acute respiratory syndrome coronavirus 2 (SARS-CoV-2) antibody efficacy and immune escape. AI-HDX fundamentally differs from the current AI tools for protein analysis and may transform protein design for various applications.

## Highlights

• A deep learning method to predict protein intrinsic dynamics from sequence
• Reveals protein dynamics information different from other machine learning tools
• High-throughput prediction for protein dynamics analysis
• Broad applications including drug discovery such as SARS-CoV-2 spike protein dynamics

## Graphical abstract

**Figure:**
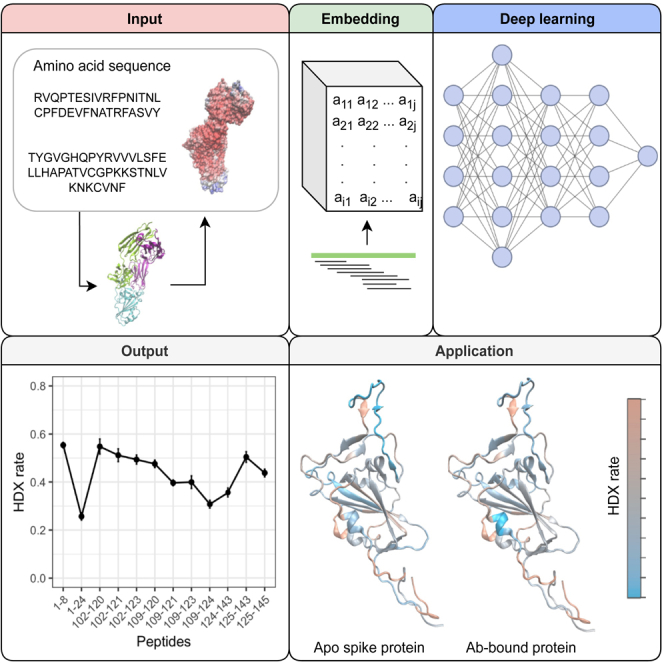

## Introduction

Protein structure prediction through computational methods has reached an epic milestone, where AlphaFold2 and RoseTTAFold can accurately predict protein backbone below 0.98 Å r.m.s.d._95_ compared to experimental structure, far more accurately than other alternative prediction methods.^1^^,^^2^ However, due to limited available tools, there is a fundamental gap in elucidating the protein structure-functional relationship through structural dynamics. The most successful experimental approaches to probe protein structure dynamics information at the amino acid residue resolution include nuclear magnetic resonance (NMR) spectroscopy, X-ray diffraction study, hydrogen-deuterium exchange (HDX) mass spectrometry (MS), and cryo-electron microscopy (EM).^3^^,^^4^^,^^5^ The computational approach, molecular dynamics (MD), simulates the physical movements of protein atoms and molecules and estimates trajectories of interacting particles in complicated systems. Although MD enables understanding molecular motions on an atomic scale, this approach has proved highly challenging for middle-size proteins and at longer time scales.^6^^,^^7^ AlphaFold2 and RoseTTAFold have successfully leveraged the rich experimental-deposited protein structure coordinates (i.e., PDB depositories), the exponentially growing genomic sequencing information, and the deep learning techniques to apply an evolutionary approach for accurate protein structure prediction. Deep learning is a class of machine learning that uses algorithms to analyze large amounts of data. These algorithms are structured in layers, allowing the system to learn from existing data and make predictions based on patterns and relationships discovered in the data. AlphaFold2 and RoseTTAFold platforms demonstrated the capacity of deep learning in extracting protein information from sequences. Recent advances in combining deep learning with cryo-EM have provided great tools to study protein dynamics information, suggesting the great utility of machine intelligence in extracting protein dynamics information.^8^ Despite all the progress, no AI-based technologies have empowered the high-throughput analysis of protein dynamics in biologically relevant environments, i.e., solution-phase dynamics and fluctuation of proteins. Such protein dynamics are often probed by NMR and HDX-MS and are essential for guiding the protein design for drug discovery, therapeutics development, biocatalyst improvement, and others.

The intrinsic dynamics of proteins are predominantly related to the protein 3D structure and the physiological conditions. From a structural perspective, the solution-phase HDX experiments present the most physiologically relevant conditions to study protein function and protein/ligand interactions,^9^^,^^10^^,^^11^ encoded by the primary amino acid sequence.^12^ The flexibility of protein structures is essential to their functions. Large-scale protein dynamics indicate protein conformational changes upon folding or ligand binding.^13^ HDX-MS is an analytical approach to measuring the large timescale protein dynamics, which relies on the protein 3D structure and its interaction with the solvent. Thus, the primary, secondary, and tertiary structures all play important roles in the protein solution-phase structure dynamics. Despite the importance, NMR is more sensitive to measure small proteins (<50 kDa) HDX due to the loss of detection sensitivity for large proteins.^14^ HDX-MS can study proteins or protein complexes with no size limits. MS for probing protein dynamics through HDX is one of the fastest methods, and the throughput can be further accelerated with automation systems.^15^ However, the experiments require purified proteins, which can be challenging to obtain. The deep learning of existing HDX-MS data to predict physiologically relevant protein dynamics will significantly improve the throughput of the analysis, empowering the protein design for broad applications.

In this study, we develop the first artificial intelligence (AI)-based approach to predict the protein dynamics information through HDX rate modeling. The AI-based HDX (AI-HDX) is fundamentally different from the MD approach and other current HDX prediction models that rely heavily on interaction algorithms and molecule physical movement prediction. We integrated experimental HDX data with the evolutionary approach, the protein 3D structure, and deep learning algorithms through modular sequence alignment, 3D folding prediction, and a deep neural network for HDX exchange rate prediction. AI-HDX successfully estimates the HDX rate in the most commonly measured range (0.2–0.7) for two testing proteins and assigns a confidence index to guide end users in interpreting the predicted HDX rates. It is worth mentioning that the HDX prediction is complimentary to MD simulation in terms of timescales, as it could probe protein structure dynamics at minute-to-hour timescales. MD is superior in providing dynamic information at sub-second timescales for fast conformational changes. Thus, the accurate and sophisticated AI-HDX holds significant potential in deciphering protein dynamics and functions, empowering protein design.

## Results

### Database curation

The training datasets for AI-HDX were collected from two HDX-MS databases, PRIDE and MassIVE.^16^^,^^17^ With experimental replicates, 63 HDX result tables were obtained from 52 proteins across 11 species and 39 known protein families (Figure S1A and Table S1). Each result table contains the experimental HDX rates for individual peptides generated from HDX-MS. We compared the sequence similarity of 4,443 peptides in a t-distributed stochastic neighbor embedding (t-SNE) plot (Figure S1B). The peptides showed a wide variation even though many belong to the same protein family, suggesting that our training data had a large diversity. The distribution of experimental HDX rates showed that more than 80% of peptide fragments have HDX rates between 0.2 and 0.7, resulting in an imbalanced training dataset (Figure S1C).

### HDX rate prediction models

**Figure 1:**
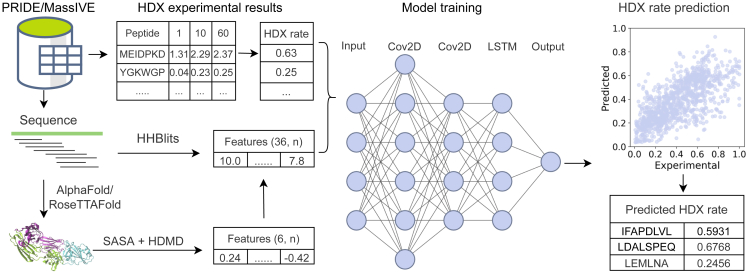
The design of the AI-HDX prediction model HDX experimental data were collected from PRIDE and MassIVE databases randomly split into the training set and the validation set. Each protein sequence was embedded by features consisting of multiple sequence alignment (MSA), solution-accessible surface area (SASA), and amino acid properties extracted from high-dimensional molecular data (HDMD). The deep learning model with two layers of convolutional neural networks (Cov2D) and long short-term memory (LSTM) neural network was trained by training sets and validation sets. Prediction of the HDX rate for each peptide from a protein sequence was reported in the output table. See also Figures S1 and S2 and Table S2.

The convolutional neural network showed outstanding performance on various tasks including protein structure predictions and membrane protein family predictions.^18^^,^^19^^,^^20^ We firstly trained a deep learning neural network (DNN) with amino acid sequence encoding by multiple sequence alignment against Uniprot (Uniref. 30_2020_06). It showed a statistically significant correlation between predicted and experimental HDX rates, with rho equaling 0.32 (Figure S2C). Although the DNN model is slightly better than the k-nearest neighbor (KNN) model, both correlation coefficients are low (Figures S2A and S2C). The sequence pattern alone may not be enough to study the protein dynamics. The protein dynamics in the solvent are related to the residue properties such as polarity, charge, and spatial location in the 3D structures.^11^ We then added the features of amino acid properties and residue solvent accessibility, accessed by high-dimensional molecular data (HDMD) of amino acid properties^21^ and the residue solvent accessible surface area (SASA) computed from 3D structures to train the models. As the 3D structures of many proteins in the training data have not been fully resolved, AlphaFold2^1^ and RoseTTAFold^2^ were used to predict the protein 3D structures for the SASA calculation. The root-mean-square error (RMSE) of the KNN model improved from 0.25 to 0.24 after adding HDMD and SASA features, with rho increasing from 0.12 to 0.16 (Figure S2B). Compared to KNN, the performance of DNN improved more significantly after including HDMD and SASA, where RMSE decreased from 0.24 to 0.17 and spearman correlation coefficients increased to 0.7134 (Figures S2C and S2D). Therefore, we selected the improved DNN as our final prediction model, AI-HDX (Figure 1). Our results indicated that the protein SASA served as an important parameter in HDX modeling to predict exchange rates.

### Validation with experimental data

Estrogen receptors (ERs) are nuclear receptors that transduce estrogen signals essential to the growth and development of a wide range of tissues.^22^ Two main ERs, ERɑ and ERβ, typically localized in the nucleus and occasionally functioning in the cytoplasm/membranes,^23^ have some overlapping functions yet different expression patterns, playing different roles in the estrogen signaling pathways.^24^^,^^25^ ERβ also acts as a tumor suppressor and as a potential drug target in cancer treatments of various human cancers.^26^ Understanding the protein dynamics of ERβ can guide designing cancer drugs that bind with ERβ. Our previous research has established that the structural dynamics of ERβ can be processed by multivariate models to predict the effects of tamoxifen or raloxifene-type effects of unknown chemical compounds with similar chemical scaffolds.^27^ Similarly, various phyto- and myco-estrogens also induce differential structure dynamics changes.^28^ The prediction of solution-phase structure dynamics of nuclear receptors like ER with AI will significantly improve drug discovery throughput. Endo-beta-1,4-xylanase (from *Trichoderma longibrachiatum* EC 3.2.1.8) is one of the enzymes required for complete hydrolysis of xylem,^29^ a plant cell wall hemicellulose component. Bacterial and fungal xylanases have been widely used in the food industry,^30^ the pharmaceutical industry,^31^ and detergents.^32^ HDX rates of human ERβ and endo-beta-1,4-xylanase (from *T. longibrachiatum*) were obtained experimentally in previous studies.^33^^,^^34^ The prediction of enzyme dynamics in a physiologically relevant environment could guide the biocatalyst design.

**Figure 2:**
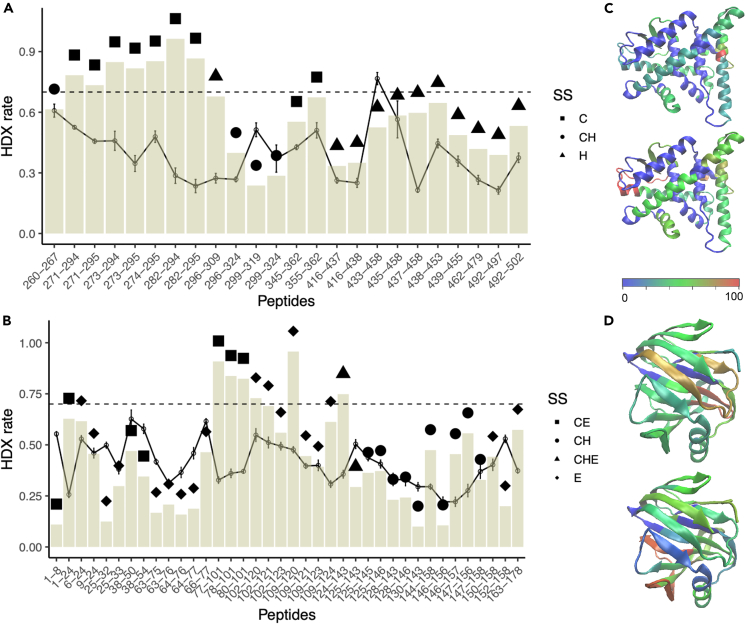
HDX prediction of ERβ and xylanase (A and B) ERβ (A) and XYN1 (B) HDX prediction, where the experimental HDX rates are in the bar charts, and the predicted HDX rates are in black dots (mean ± SE). Transparency of the points indicated the prediction confidence of the HDX rates compared to the measured rates. Shapes represent the secondary structure (SS) of the peptide located. The secondary structure is coded into single letters as H - alpha-helix, E − beta-sheet, and C - loop and turn. The dashed line indicated an HDX rate of 0.7. C. ERβ-predicted (top) and experimental (bottom) HDX rates presented on the 3D structure. The structure is predicted by AlphaFold2. D. XYN1-predicted (top) and experimental (bottom) HDX rates presented on the 3D structure (PDB: 1XYN). The colorbar indicates the percentage of HDX rates.

To prove the broad applicability of the AI-HDX, the experimental HDX rates of two proteins (i.e., ERβ and endo-beta-1,4-xylanase) are compared to the AI-HDX predicted values. Our results showed that the HDX prediction in ERβ and xylanase had a similar accuracy, with RMSE of 0.25 and 0.26, respectively. We noticed that the performance of AI-HDX was reasonably accurate when the HDX rate is smaller than 0.7 (Figures 2A and 2C). The less-accurate predictions at the higher (>0.7) HDX rates or extremely low HDX rates (<0.2) (Figures 2A and 2B) might result from lacking fast- and slow-exchange peptides (>0.7 and <0.2 HDX rate) in the training dataset (Figure S1C). We further evaluated the prediction accuracy in regard to secondary structures. The predicted HDX rates were most accurate when the peptides were located in a specific secondary structure (i.e., alpha-helix and beta-sheet). However, the predicted HDX rates are less accurate when the peptides are in loops and turns (Figures 2A and 2C). Both ERβ and xylanase showed a low accuracy on the HDX-predicted rates in loops and turns (Figures 2C and 2D). For predicted 3D structures, AlphaFold2/RoseTTAFold predictions may have a higher accuracy to predict alpha-helices and beta-sheets structures than loops and turns. Expanding the training set when more experimental HDX data are available might further improve the prediction accuracy of the AI-HDX in the loop and turn regions in the future.

### Confidence index of AI-HDX

The predictions on the peptides with low (<0.2) or high (>0.7) HDX rates were less accurate than those on peptides with HDX rates within 0.2–0.7 (Figures 2A and 2C). For most peptides with experimental HDX rates in the range of 0.2–0.7, the RMSE between predicted HDX and experimental HDX rates is 0.14, while peptides with HDX rates lower than 0.2 or greater than 0.7 have an RMSE of 0.22 (Figure S3). Because the AI-HDX model is the first *de novo* model to predict the HDX rate, we defined a parameter as a confidence index (CI) to evaluate the prediction confidence for each peptide’s HDX rate. We assessed the HDX predictions of proteins in the model trained by five randomly split training and validation sets. The CI value largely decreased when the predicted HDX was more than 0.7 and dropped to zero in the range of HDX prediction between 0.9 and 1 in all five training models, suggesting a low prediction accuracy of AI-HDX for the peptides with high HDX rates (Figure S4).

**Figure 3:**
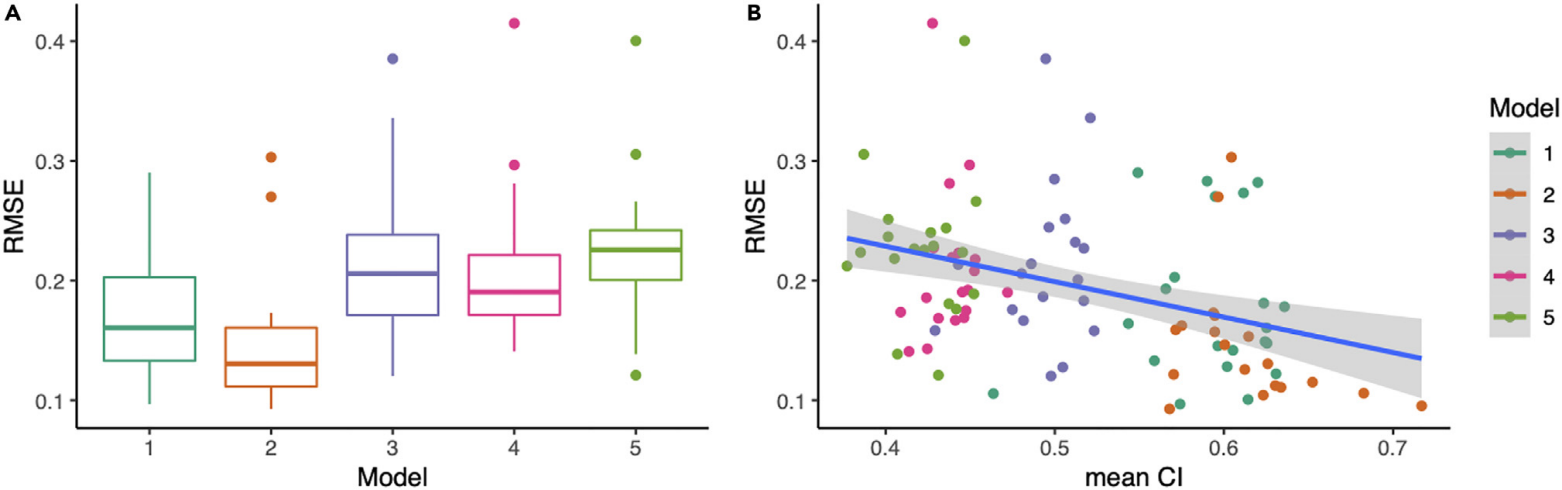
AI-HDX performance in the five randomly split validation datasets (A) Boxplot of RMSE on HDX predictions in the proteins from five training models. (B) Scatterplot of the mean CI and RMSE on HDX predictions in the proteins from five training models. See also Figure S4.

The five training models showed RMSE ranging from 0.09 to 0.42 in the proteins from validation sets. We found that the second training model showed a significantly low RMSE compared to others, indicating unbalanced training data (Figure 3A). It is necessary to evaluate the relationship between CI and RMSE. The CI from all five validation sets showed a negative correlation with RMSE. The low RMSE correlated with a high CI score, which suggested that the CI model adequately represented the prediction accuracy between prediction and experimental data (Figure 3B). We expect that the prediction accuracy and CI utility will improve when more experimental HDX data are available.

### Application on SARS-CoV-2 variant receptor-binding domain (RBD) dynamics

**Figure 4:**
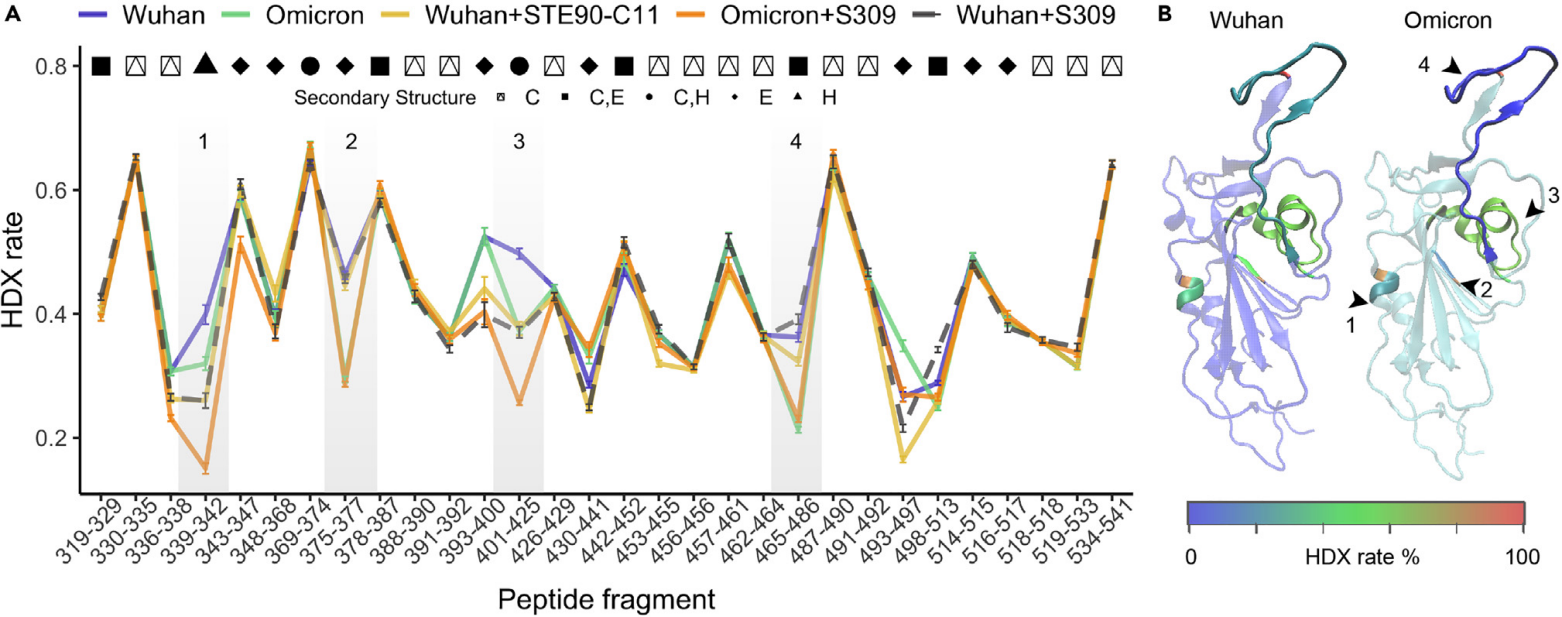
HDX prediction of SARS-CoV-2 RBD in Wuhan and Omicron variants (A) HDX rates (mean ± SE) of peptide fragments in Wuhan or Omicron variants with or without antibodies. Antibody-bound RBD complexes 3D structures were obtained from PDB structures 7B3O and 7TLY. Gray boxes indicated significant HDX rate changes between Wuhan and Omicron RBDs in the apo and binding forms. The secondary structure is coded into single letters as H - alpha-helix, E − beta-sheet, and C - loop and turn. (B) 3D structures of Wuhan and Omicron RBD predicted by RoseTTAFold. The peptide regions with significant changes between Wuhan and Omicron variants were highlighted by the predicted HDX rates with the indicated color scale. Arrows indicate the peptide locations corresponding to the gray boxes in Figure 4A.

To access the broad application of AI-HDX, we applied the AI-HDX analysis on the receptor-binding domain (RBD) of the RNA virus severe acute respiratory syndrome coronavirus 2 (SARS-CoV-2) spike protein. Recent studies have used HDX-MS to investigate the protein dynamics of the SARS-CoV-2 spike protein when it binds to the human receptor angiotensin converting enzyme 2 (ACE2) or neutralizing antibodies, providing insights into neutralization effects by spike protein-targeted antibodies.^35^^,^^36^^,^^37^ A new confirmation of prefusion spike protein called open trimer was discovered by HDX-MS,^38^ suggesting that HDX-MS is potentially useful for vaccines and drug discovery. Since the COVID-19 outbreak in early 2020, the virus has evolved rapidly and caused several pandemic outbreaks when different variants emerged.^39^ Neutralizing antibodies that block the spike protein’s binding to its cellular receptor may easily lose their binding ability as the virus mutates, but the mechanism remains elusive.^40^ To evaluate how RBD mutations would impact RBD/antibody binding, we applied AI-HDX to study the dynamics of the original Wuhan variant and Omicron variant and their RBD/antibody binding events: Wuhan RBD binding with the antibody STE90-C11 ^41^ and the Omicron variant binding with the antibody S309 ^42^^,^^43^. The 3D structures of the apo-RBD in Wuhan and Omicron variants were predicted by RoseTTAFold since the apo-RBD X-ray structure is not available. The 3D structures of the STE90-C11-binding RBD (PDB: 7B3O),^41^ S309-binding Omicron RBD (PDB: 7TLY),^43^ and S309-binding Wuhan RBD (PDB: 6WPS)^42^ were used to predict the HDX rate of the antibody-bound RBDs. STE90-C11 is an effective anti-SARS-CoV-2 antibody but loses binding to K417N-mutated Omicron variant spike protein.^40^^,^^41^ Omicron variant RBD showed decreased HDX rates in three peptide regions (375-377, 401-425, and 465-486) compared to the Wuhan variant RBD (Figure 4A). The Omicron variant RBD has five mutations, S375F, K417N, S477N, T478K, and E484A mutations, which are involved in the binding interactive surface of the antibody STE90-C11 ^41^. The Omicron-mutated regions showed decreased HDX rates compared to those of the Wuhan variant regions, where the Wuhan variant interacts with STE90-C11 (Figure 4A). The reduced HDX rates suggested that the Omicron RBD may be less dynamic than the original Wuhan RBD, in which the mutations have stabilized the RBD. The Omicron RBD might have presented at a more thermodynamically favored status, which leads to antibody STE90-C11 being unable to bind with it due to unfavorable Gibbs free energy for the binding interaction (Figure 4B). More importantly, the binding affinity is also related to the accessibility of the spike protein epitopes. The more dynamic the epitopic peptides are, the more interaction with the solution phase will be and the easier they are recognized and bound by the neutralizing antibodies.^44^ The low HDX rates in Omicron RBD peptides may have decreased the presentation of the epitopic peptides to STE90-C11, which explains why Omicron RBD can escape STE90-C11 responses. From a broader point of view, the three stabilized regions may help to explain the immune escape of Omicron in general.

In contrast, antibody S309 is able to bind Omicron RBD^43^ and Wuhan RBD (PDB: 6WPS).^42^ The AI-HDX profiles revealed that two peptides 339-342 and 393-425 (where the RBD mutations are present) had lower HDX rates in the S309-bound Omicron RBD than in the Omicron apo-RBD (Figure 4A). Different from the STE90-C11, antibody S309 recognizes epitopic peptide 337-344 ^42^, where HDX rates did not show a significant difference between Omicron and Wuhan variants. However, we predicted HDX rate reduction in the amino acid 337-344 region when S309 binds with the Omicron RBD (Figure 4A). The favorability of the S309 epitope and Omicron RBD interaction is consistent with the reduced predicted HDX rates in the S309-bound Omicron RBD, compared to those of the STE90-C11/Wuhan RBD interaction complex. This suggests that, even though the Omicron RBD is more stabilized than the Wuhan variant, S309 and Omicron RBD interaction is still thermodynamically favored. In agreement with AI-HDX predictions, the structural dynamics analysis from elastic network models showed changes in the dynamics cross-correlation maps between Wuhan and Omicron variants, which suggested a reduction in protein structural dynamics in Omicron RBD (Figure S5). Interestingly, the predicted HDX rates of the Wuhan RBD+S309 closely align with Omicron+S309 and Wuhan RBD+STE90-C11, where peptides 337-347, 392-429, and 493-497 of the Wuhan RBD had reduced HDX rates upon S309 binding when compared with the apo-RBD. (Figure 4A). However, in region 4, the predicted HDX rate increased slightly in the Wuhan+S309 complex compared with the apo-Wuhan RBD, which could be due to model prediction uncertainty. In summary, the important RBD/antibody interaction regions highlighted that the structure dynamics could play a critical role in antibody/antigen binding and recognition. The AI-HDX can potentially be applied to estimate the binding affinity of the available antibody drugs to future COVID-19 RBD variants.

### Advantages of *de novo* AI-HDX

The AI-HDX model presents as the first *de novo* machine learning model that solely relies on protein sequences and predicted 3D structures (i.e., AlphaFold2 and RoseTTAFold predictions) to estimate HDX rates for apo-proteins. The model is able to encode structural information from protein-protein complexes in order to predict HDX rates for protein-protein interaction events. However, 3D structures are required. Different from the AI-HDX, previous studies (HDXmodeller and PyHDX) have focused on using machine learning to optimize the available experimental HDX data and improve the HDX resolution, where residue-level protection factor information can be obtained.^45^^,^^46^ In contrast, AI-HDX predictions are solely data driven, generating amino residue-level HDX information through computational random digestion. The traditional HDX experiments are limited by enzyme choices for digestion and quick digestion time, which inevitably leads to limited information on amino acid resolutions. The AI-HDX took advantage of the existing HDX database, compared to HDXmodeller and PyHDX, in two aspects: first, the HDX prediction by AI-HDX is primarily based on sequence and structural information as the experimental HDX data of the same protein are not required, which could significantly facilitate structure dynamics prediction; secondly, AI-HDX can predict HDX rates for fragments randomly dissected from a protein, even at a single amino acid length, without the real experimental HDX. As a demonstration, we split a peptide fragment ‘ISVRNSPRTSGTVTVQNHFNAW’ (residues 125-146) from xylanase into different lengths and predicted their HDX rates. As one of the longest fragments in the testing dataset, it can produce more cleaved peptides for analysis. AI-HDX was able to predict the HDX rates for each fragment subsetting from the peptide (Figure S6). The controllable peptide digestion pattern can thus present a superior advantage to the experimental HDX analysis.

## Discussion

This work provides a proof of concept that the deep learning technique can predict the HDX rate of peptides in HDX-MS experiments. Our previous work established the experimental method that HDX-MS measurements of ERs can be used to classify ER modulators like tamoxifen and raloxifene.^27^^,^^33^ AI-HDX will allow us to survey a broad range of drug targets. Together with docking, the platform has the potential to classify large chemical/drug libraries and facilitate protein engineering and therapeutic strategies. Here we have exhibited AI-HDX utility in predicting COVID-19 spike protein variant dynamics and the potential to identify the effective antibody treatment. Unlike the deep learning model predicting ACE2-RBD binding and antibody escape through sequence mutations,^47^ AI-HDX predicts protein structural dynamics to explain the potential antibody escape. Other promising applications could include analyzing important regions in protein/substrate interaction and guiding site mutagenesis for enzyme engineering. Compared with HDX experiments, the AI-HDX thus presents several advantages: rapidity, requiring minimum resources, and the ability to reach a single amino acid resolution. The AI-HDX prediction provides significant benefits to guide protein engineering, yet there are several considerations to improve the AI-HDX model.

First, the performance of the current model depends on the available HDX datasets. A limited amount of training samples (experimental HDX data) restricted the learning depth of the DNN model. Currently, we are only able to retrieve 63 good-quality HDX datasets in the literature. The curated training dataset contains a small number of peptides with HDX rates <0.2 and from 0.7 to 1, which leads to a less-optimal prediction in those two ranges. For example, we performed AI-HDX analysis on apo-RBD and ACE2-bound RBD to compare whether the predicted HDX rate for protected regions is correlated with experimental HDX-MS.^37^ We observed a good agreement between experimental and predicted HDX rates for peptides with exchange rates between 0.2 and 0.7 (Table S3). As a matter of fact, for peptides 421-431, 432-449, and 490-510, our prediction of the HDX rates is quite consistent with the measured HDX rates: 0.28 versus 0.22, 0.49 versus 0.55, and 0.49 versus 0.44, respectively, with the latter value being the measured HDX rates. However, AI-HDX failed to predict the decrease in HDX rates in the ACE2-bound peptides for the two protected regions (Table S3). We found that the two peptides had high exchange rates (>0.7), which fell within the inaccurate range. These highly exchanged peptides are in turns and coils, highlighting the necessity to examine the AI-HDX-predicted rates and the corresponding protein secondary structures. We expect this can be improved when more HDX rates on high exchangeable peptides are available. Second, future improvement of protein 3D structure prediction will help to improve the AI-HDX prediction. For example, regions (i.e., loops and turns) that are hard to characterize in the X-ray crystallography may also impact the accuracy of AI-HDX prediction as the input layer accuracy is impaired, which may lead to relatively low prediction accuracy. We observed that protein structure predictions at some regions (e.g., loops and turns) with low HDX accuracy showed lower prediction confidences by AlphaFold2 as well (Figure S7). Conversely, the HDX prediction at alpha-helices or beta-sheets, where protein structure predictions are highly confident, achieved high prediction accuracy (Figure 3). Third, standardization of the HDX community experimental practice would improve the database quality and AI-HDX model precision. The inherent measurement uncertainty in the curated database is hard to estimate due to the lack of interlaboratory HDX measurement precision characterization. For example, for a specific peptide, the HDX rate measurement could vary in different laboratories. It is hard to estimate the precision and repeatability of each reported HDX rate in the curated database as the HDX experiments were performed in individual laboratories. An interlaboratory comparison of HDX analysis on the same protein and the same protocol has reported noticeable interlaboratory variations.^48^ Hudgens et al. showed that for the same peptide, the measured exchange rate may range from 70% to 100%.^48^ As for the interlaboratory reproducibility, the standard deviation (four peptide sets with 258 peptides) ranges from 6.5% to 22.3%, considering the temperature and exchange time variations. It is thus reasonable to estimate that comparable or even bigger measurement variations exist in the curated HDX database (63 proteins including 4,443 peptides) measured by 16 different laboratories (Table S1). Of the 4,443 peptides curated, 2,121 (47.7%) were from studies with exchange time points shorter than 1 h, which is the time point of interest for our analysis. In addition, 648 peptides had HDX rates within the range of <0.2 and >0.7, which may introduce variations in the training model that lead to prediction error. Standardization of HDX procedures could minimize the variations^10^ and thus improve the data quality and the AI-HDX prediction accuracy.

The prediction accuracy of the AI-HDX model is largely dependent on the structural information. In the xylanase dataset, we found a higher prediction error for short peptides with residues less than 10 amino acids (i.e., an RMSE of 0.26 between predicted and experimental HDX rates). For larger peptides with more than 10 amino acids, the RMSE values were smaller than 0.2 (Figure S8). We expect this is reasonable as most of the training data contain peptides longer than 10 residues (Figure S9), and longer peptides sequence may render more reliable structural predictions. AI-HDX can predict HDX rates at the residue level, but the accuracy of these predictions is uncertain. Most peptides in the training dataset are within a certain length range (Figure S9). The long short-term memory (LSTM) algorithm used by AI-HDX is designed to learn the context and patterns of protein sequences. Protein structures, such as loops, bridges, helices, and sheets, typically consist of more than two residues. Predicting HDX rates at the residue level provides high resolution, but it may also introduce errors due to a lack of training data at that resolution. With the growth of HDX data and the development of residue resolution HDX analysis, AI-HDX has the potential to learn and accurately predict single residue HDX rates.

Overall, we have established the first *de novo* deep machine learning model to predict apo-protein structure dynamics directly from amino acid sequences. We illustrated the model utility using SARS-CoV-2 RBD and its variants interacting with antibody recognition. Our AI-HDX analysis of SARS-CoV-2 RBD and its variants suggested that the variant structure dynamics change may play an essential role in antibody recognition, thus potentially providing strategies for COVID antibody treatment. We believe that in the future, the AI-HDX could accelerate data-driven protein dynamics characterization to study protein-protein interaction, which can guide future drug discovery, protein design and engineering, enzyme dynamics, and essential mechanism studies. Our study provides a strategy to leverage experimental data, deep learning modeling, and data science to address fundamental questions in protein biochemistry and biophysics.

### Limitations of the study

The current AI-HDX model has several limitations. Firstly, the performance of AI-HDX is dependent on the quality and availability of HDX datasets. With only 63 public HDX datasets curated and less than 25% of peptides with HDX rates greater than 0.7 or smaller than 0.2, the learning depth and prediction accuracy on the high or low dynamic regions are compromised. Secondly, AI-HDX is not a standalone program and relies on protein 3D prediction from other tools such as AlphaFold2 or RoseTTAFold, which may affect its accuracy. Finally, there is a need for standardization of the HDX experiment practices and data-reporting format, which may help improve the prediction accuracy of AI-HDX. The standardization of the experimental practices will help reduce the HDX data variation across different laboratories. The characterization of interlaboratory HDX measurement precision and a consistent data-reporting format can facilitate curation of the HDX database and improve the reliability of AI-HDX predictions.

## STAR★Methods

### Key resources table

**Table:** 

REAGENT or RESOURCE	SOURCE	IDENTIFIER
**Chemicals, peptides, and recombinant proteins**		

Endo-1,4-β-Xylanase M3 (*Trichoderma longibrachiatum*)	Megazyme	E-XYTR3

**Deposited data**		

HDX-MS datasets	Proteomics Identifications Database	https://www.ebi.ac.uk/pride/
HDX-MS datasets	Mass Spectrometry Interactive Virtual Environment	https://massive.ucsd.edu/ProteoSAFe/static/massive.jsp
Protein sequences	UniProt	https://www.uniprot.org/
1XYN, 6WPS, 7B3O, 7TLY	Protein DataBank	https://www.rcsb.org/
Omicron spike protein mutations	Outbreak.info	https://outbreak.info/

**Software and algorithms**		

HHBlits	Remmert et al.^49^	https://github.com/soedinglab/hh-suite
DSSP	Kabsch and Sander.^50^	http://swift.cmbi.ru.nl/gv/dssp/
AlphaFold2	Jumper et al.^1^	https://github.com/deepmind/alphafold
RoseTTAFold	Baek et al.^2^	https://github.com/RosettaCommons/RoseTTAFold
HDXanalyzer	Sun et al.^51^	
PeptideMass	Wilkins et al.^52^	https://web.expasy.org/peptide_mass/
VMD v1.9.4	Visual Molecular Dynamics	https://www.ks.uiuc.edu/Research/vmd/
Python 3.8	Python Software Foundation	https://www.python.org
R software v4.1.3	The R Project for Statistical Computing	https://www.r-project.org

### Resource availability

#### Lead contact

Further information and request for resources should be directed to and will be fulfilled by the lead contact, Susie Y. Dai (sydai@tamu.edu).

#### Materials availability

This study did not generate new unique reagents.

### Method details

#### HDX data processing

We collected publicly available processed HDX-MS results tables from in-house HDX experiments, PRIDE, and MassIVE databases (Table S1). Data from apo-proteins at 1 h were selected to develop this model. 21 out of 63 datasets from 53 proteins have the longest data point within an hour (either 30 or 50 min), we then used the longest time point instead of the 1-h time point. 28 out of 63 datasets, including 21 proteins and 2,121 peptides (47.7% of total) have the longest data points at either 30 or 50 min. The HDX rates at the selected time points were calculated with a back-exchange rate of 70% correction. The 70% correction factor is a rough averaged estimation based on historical data and recommendations for the best HDX practice.^10^^,^^48^^,^^51^ To train the machine learning models, the HDX rates were represented as deuterium ratios between 0 and 1.

#### Protein sequence embedding

Proteins were embedded with the multiple sequence alignment (MSA) approach. Protein primary sequences were obtained from Uniprot as indicated IDs in Table S2. The primary sequences were aligned against Uniprot Reference Clusters (Uniref. 30_2020_06) by HHblits^49^ and each amino acid was encoded with a vector composed of 30 numbers, indicating evolutionary similarities.^53^ Each vector was scaled into a uniform distribution following the encoding methods by Liu et al. (2020) with a sigmoid function.^54^ To include the amino acid features, amino acids were indexed with multi-dimensional molecular data (HDMD) for amino acid properties^21^ and reduced dimension to five by principal component analysis. Residue solvent accessible surface area (SASA) was found to correlate with HDX.^55^ We therefore calculated residue SASA by DSSP^50^ using protein 3D structures.^56^ All proteins were embedded with a 36 x N array (N is the protein sequence length).

Protein 3D structure is usually missing two termini due to the difficulty of crystallization. To calculate the SASA of each amino acid for full length, protein 3D structures were predicted by the latest machine learning prediction algorithms, AlphaFold2 and RosettaFold.^1^^,^^55^ AlphaFold protein structure database (https://alphafold.ebi.ac.uk/) provides predicted 3D structures for proteins from 21 model organisms. Our training data obtained protein 3D structures from model organisms from the AlphaFold database. Proteins from non-model organisms were predicted using RosettaFold, which has similar accuracy as AlphaFold but is less computationally intensive than AlphaFold.

#### Machine learning models to predict HDX rates

Two models were constructed to perform the HDX rate prediction: 1) a k-nearest neighbor (KNN) algorithm assuming that peptides with similar sequence information showed a close rate of H/D exchange; 2) a deep neural network (DNN) algorithm with the assumption that residue composition and their relationships are the key components of determining H/D exchange (Figure 1). We randomly split the 62 protein datasets into 70% of the data as the training set and 30% as the validation set. The two models were trained and validated by the same training and validation dataset. We used Spearman’s correlation coefficient (rho value) and root-mean-square error (RMSE) to compare the predicted HDX rates and experimental HDX rates for model evaluation.

#### K-nearest neighbors (KNN) model

We tried different machine learning algorithms to learn the peptide patterns and predict the HDX rates. To start with a simple model, we used the KNN algorithm with k equal to 10 to predict the HDX rates from input peptides. The KNN model predicted the HDX rate of a peptide based on the 10 known peptides showing the closest features to it.

#### Neural network model

We constructed a 2D convolutional neural network (CNN) coupled with a bidirectional recurrent neural network long-short term memory (biLSTM) model to learn the HDX rates of digested peptides. The 2D convolutional block consists of two 2D CNN layers with ReLu activation, kernel regularization of 0.0001, max pooling, and dropout. The recurrent neural network block has one LSTM layer with TanH activation and dropout. The dense block consists of one dense layer with linear activation, one dense layer with ‘Softplus’ activation, and the output layer with sigmoid activation. The binary cross-entropy loss function was employed in the training model, and we used the ‘Adam’ optimizer with a 0.01 reduced learning rate for each epoch to update the model weights. A total of 55,567 trainable parameters were generated. The 63 HDX datasets were randomly split into training data which contains 70% of the entire dataset, and validation data which contains the rest of the 30% dataset. The model was trained for 100 epochs to reach a validation mean square error smaller than 0.3.

#### Prediction score implementation

Due to the limited number of publicly available HDX data, we calculated a confidence index for each of the predicted HDX rates, indicating a probability that the predicted HDX rate truly represents the actual rate. Hudgens et al. (2019) reported that the reproducibility of HDX-MS from different laboratories is (0.9 ± 9) %.^48^ Therefore, we considered the HDX prediction to be accurate if it has less than a 10% difference from the experimental HDX rate. We split the validation dataset including 1,057 peptides into ten equal intervals with a 0.1 difference (i.e., HDX rate 0–0.1, 0.2–0.3 etc) based on their measured HDX rates. For each interval, the confidence index (CI) is calculated by the number of correctly predicted peptides divided by the total number of peptides (Equation 1):

(Equation 1) $$CI=\frac{1}{N}\sum f\left(n\right)$$

(Equation 2) $$f\left(n\right)=\left\{ \begin{array}{c} 1,\left|\hat{y}-y\right|\leq 0.1\\ 0,\left|\hat{y}-y\right|\geq 0.1 \end{array}\right.$$

CI is given to the prediction when the predicted HDX ratio sits within a specific interval. For example, suppose the predicted HDX rate for a peptide is 0.22. In that case, the prediction CI is calculated based on the number of correctly predicted peptides divided by the total number of peptides in the experimentally measured HDX range of 0.2–0.3 in the training dataset.

#### Prediction of SARS-CoV-2 RBD spike protein HDX rates

The sequence of SARS-CoV-2 RBD spike protein was amino acid 319 -541 from Uniprot ID P0DTC2. Omicron spike protein mutations were obtained from the public domain: https://outbreak.info/. Both original Wuhan RBD and Omicron RBD were embedded following the same protein sequence embedding method described above. The SASA for each amino acid in apo-RBD was obtained from the 3D structure predicted by RoseTTAFold. The SASA in antibody-bound Wuhan RBD was obtained from the X-ray crystal structure of RBD and STE90-C11 Fab complex (PDB: 7B3O).^41^ The SASA in antibody-bound Omicron RBD was obtained from the cryo-EM structure of B.1.1.529 Omicron RBD and S309 Fab (PDB: 7TLY).^43^ The SASA in S309-bound Wuhan RBD was obtained from the cryo-EM structure of the spike protein and S309 Feb complex (PDB 6WPS).^42^ The peptide fragments were generated by *in silico* protease digestion using the online tool PeptideMass with pepsin (pH 1.3).^52^ The HDX rates of apo-RBD and holo-RBD peptide fragments were predicted by 10 AI-HDX models and the average of 10 predicted HDX rates was visualized in 3D structures as the indicated color bar by VMD-1.9.4a55.^57^ Theoretical structural dynamics analysis was performed using elastic network models (http://enm.pitt.edu/) with two antibody-bound RBD structures.

### Experimental model and subject details

#### HDX experiments of XYN I and ER

The wild-type (WT) XYN I used in this study was from *T. longibrachiatum* purchased from Megazyme (Megazyme International Ireland Ltd., Wicklow, Ireland). The purified proteins used in the HDX experiments were conducted as previously described.^34^ Estrogen receptor beta was analyzed and reported previously.^33^

### Quantification and statistical analysis

#### Analysis of XYN I experimental HDX data

The mass spectra were analyzed as previously described^34^ by HDXanalyzer,^51^ with a back-exchange rate of 70% and accounting for the solution buffer of 80% deuterium.

#### Validation of prediction models

As a regression model, the model performance was evaluated by the root mean standard error. Spearman’s correlation coefficient determined the coefficient of predictions and experimental HDX rates. Statistical dependence was determined by hypothesis testing with a p value less than 0.05, using the R function `cor.test`.

## Data Availability

The paper analyzes existing, publicly available data. These accession numbers for the datasets are listed in this paper’s supplemental information and in the key resources table. All original code has been deposited at https://github.com/Environmentalpublichealth/AI-HDX, and is publicly available as of the date of publication. DOIs are listed in the key resources table.
