# Supplementary material for: Artificial intelligence-based HDX (AI-HDX) prediction reveals fundamental characteristics to protein dynamics: Mechanisms on SARS-CoV-2 immune escape
Source: iScience. 2023 Feb 27;26(4):106282. doi: 10.1016/j.isci.2023.106282 (PMC9968663; doi:10.1016/j.isci.2023.106282)
Supplement: Document S1. Figures S1–S9 and Tables S1–S3 [file mmc1.pdf]

iScience, Volume *26*

Supplemental information

Artificial intelligence-based HDX (AI-HDX) prediction reveals fundamental characteristics to protein dynamics: Mechanisms on SARS-CoV-2 immune escape

Jiali Yu, Ugur Uzuner, Bin Long, Zachary Wang, Joshua S. Yuan, and Susie Y. Dai

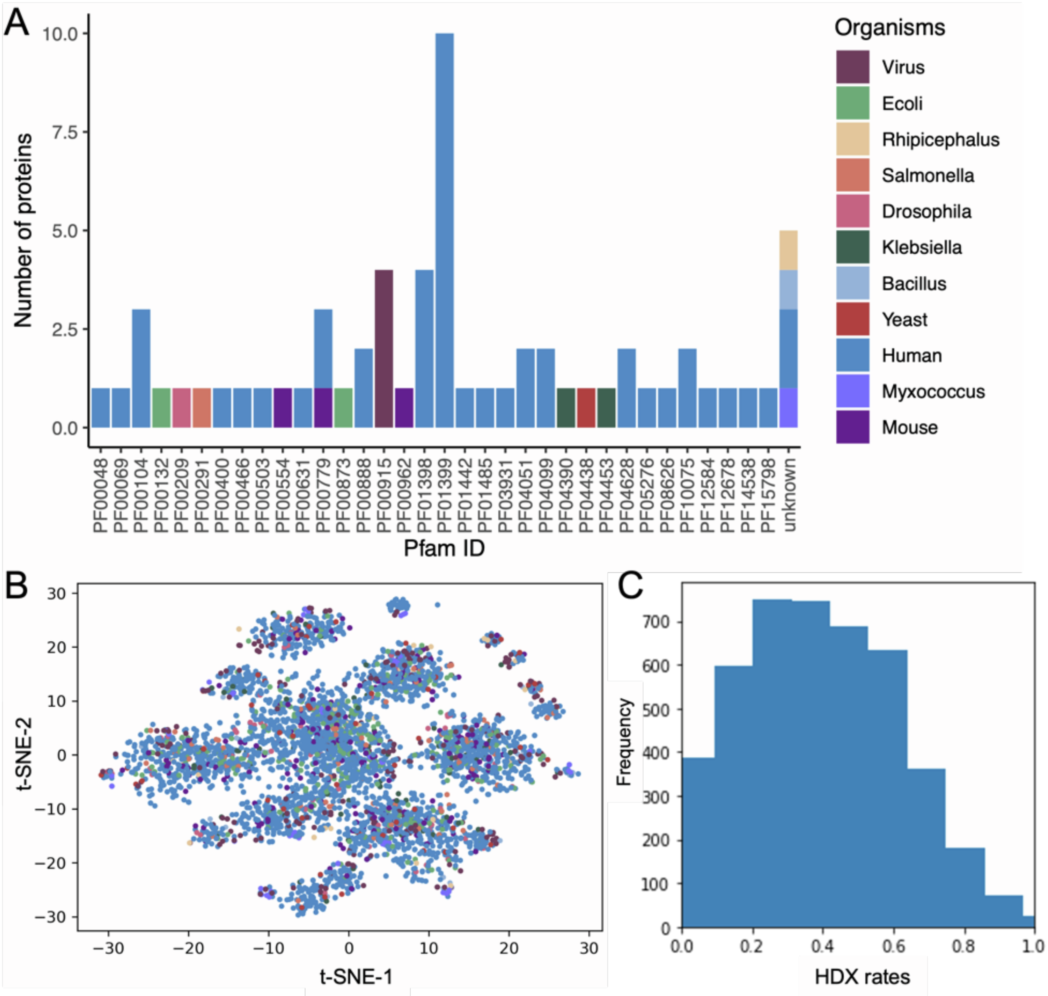

Figure S1. A. Overview of collected HDX datasets. Related to STAR Methods and Figure 1.

A. The origin of proteins used as training data. Protein families are denoted by Pfam IDs. Pfam descriptions were listed in Supplementary Table 2. B. t-SNE plot visualized the distribution and similarity of peptides in training data. C. Histogram of HDX rates in individual peptides from all 62 datasets that were used in training data.

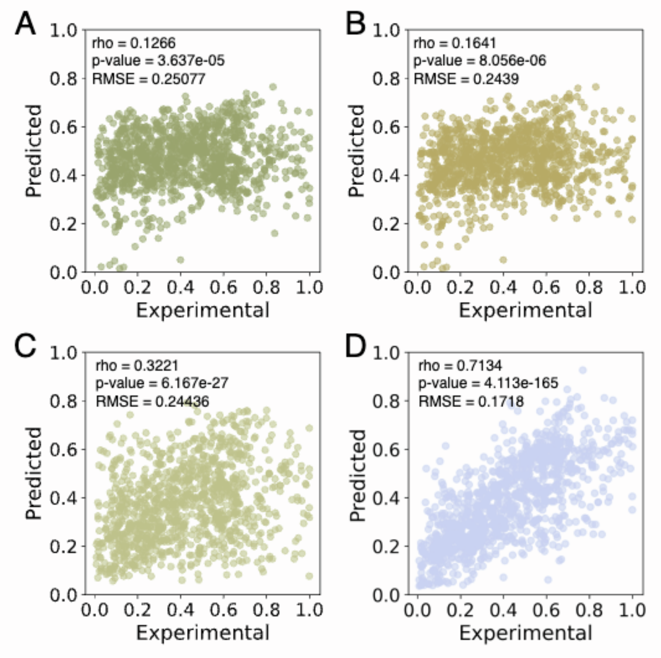

Figure S2. Scatter plots of predicted and measured HDX rates. Related to Figure 1.

K-nearest neighbor model (A) and neural network model (C) with MSA only. K-nearest neighbor model (B) and neural network model (D) with MSA and SASA. The rho value indicates Spearman's correlation coefficient. The p-value determines the statistical significance of the correlation. RMSE indicates the prediction errors between predicted and measured HDX rates, a lower RMSE means a better prediction accuracy.

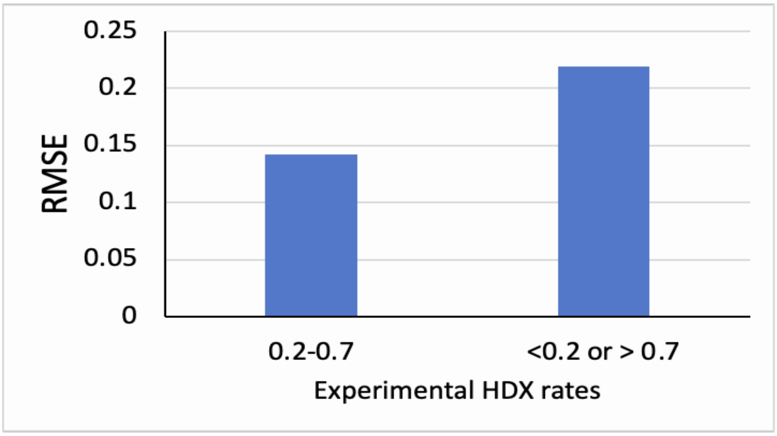

Figure S3. RMSE of predicted and experimental HDX rates from peptides in validation and testing data. Related to STAR Methods.

The experimental HDX rates were divided inside the range of 0.2-0.7 and outside the range of 0.2-0.7. RMSE between AI-HDX predicted rates and experimental HDX rates were computed.

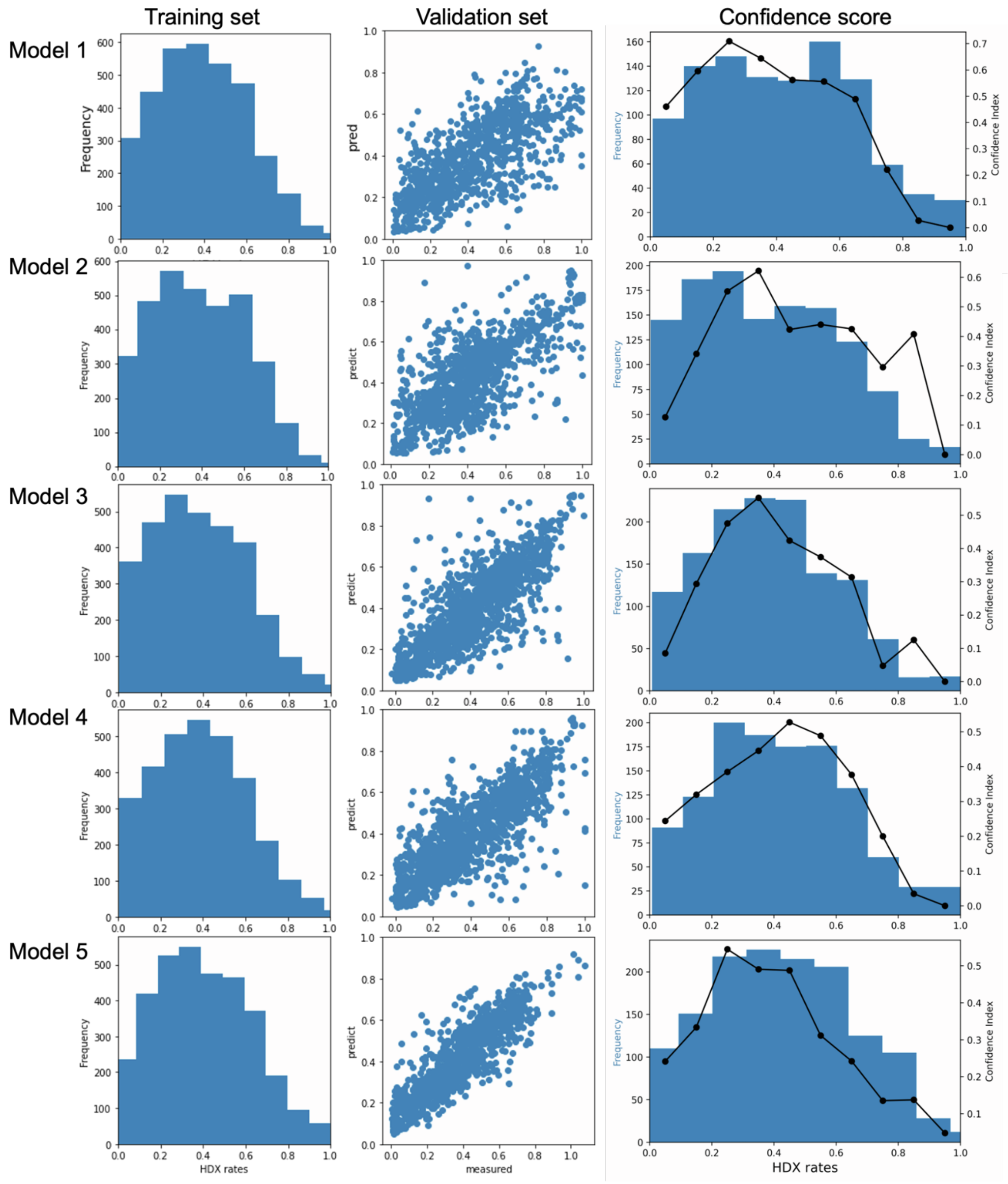

Figure S4. AI-HDX model trained by five randomly split training sets and validation sets. Related to STAR methods and Figure 3.

For each training model, we presented the distribution of training labels (experimental HDX rates in the training set), the correlation between measured and predicted HDX rates from the validation set, and confidence scores. The confidence index (CI) was calculated in 10 HDX intervals, comparing the predicted HDX and experimental HDX rates.

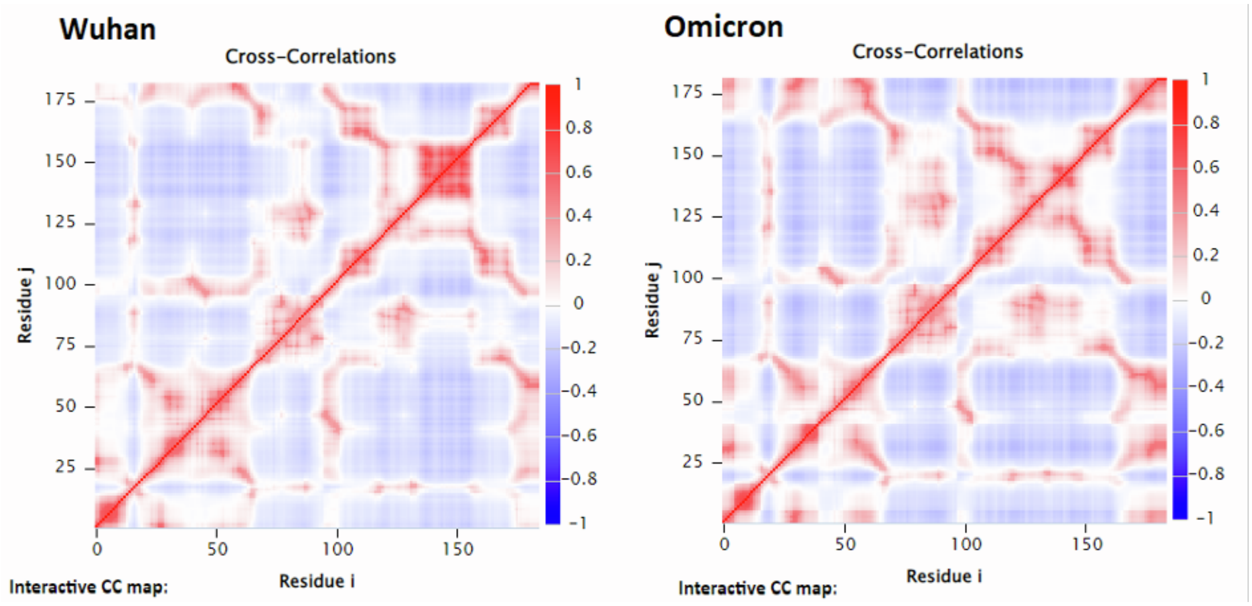

Figure S5. Intramolecular biochemical bond distribution contact maps for Wuhan RBD (left, 7B3O) and Omicron RBD (right, 7TLY). Related to Figure 4.

Cross-correlations between each residue are calculated by Pearson's correlation coefficient. Positive values indicated the residue pairs were correlated, while negative values indicated the residue pairs were anti-correlated.

125	125	I	1	0.44
125	126	IS	2	0.17
125	127	ISV	3	0.16
125	128	ISVR	4	0.4
125	129	ISVRN	5	0.63
125	130	ISVRNS	6	0.56
125	131	ISVRNSP	7	0.43
125	132	ISVRNSPR	8	0.32
125	133	ISVRNSPRT	9	0.42
125	134	ISVRNSPRTS	10	0.45
125	135	ISVRNSPRTSG	11	0.52
125	136	ISVRNSPRTSGT	12	0.33
125	137	ISVRNSPRTSGTV	13	0.46
125	138	ISVRNSPRTSGTVT	14	0.45
125	139	ISVRNSPRTSGTVTV	15	0.46
125	140	ISVRNSPRTSGTVTVQ	16	0.56
125	141	ISVRNSPRTSGTVTVQN	17	0.57
125	142	ISVRNSPRTSGTVTVQNH	18	0.5
125	143	ISVRNSPRTSGTVTVQNHF	19	0.44
125	144	ISVRNSPRTSGTVTVQNHFN	20	0.44
125	145	ISVRNSPRTSGTVTVQNHFNA	21	0.41
125	146	ISVRNSPRTSGTVTVQNHFNAW	22	0.41
126	146	SVRNSPRTSGTVTVQNHFNAW	23	0.42
127	146	VRNSPRTSGTVTVQNHFNAW	24	0.33
128	146	RNSPRTSGTVTVQNHFNAW	25	0.32
129	146	NSPRTSGTVTVQNHFNAW	26	0.27
130	146	SPRTSGTVTVQNHFNAW	27	0.29
131	146	PRTSGTVTVQNHFNAW	28	0.37
132	146	RTSGTVTVQNHFNAW	29	0.27
133	146	TSGTVTVQNHFNAW	30	0.24
134	146	SGTVTVQNHFNAW	31	0.16
135	146	GTVTVQNHFNAW	32	0.23
136	146	TVTVQNHFNAW	33	0.12
137	146	VTVQNHFNAW	34	0.16
138	146	TVQNHFNAW	35	0.16
139	146	VQNHFNAW	36	0.21
140	146	QNHFNAW	37	0.11
141	146	NHFNAW	38	0.18
142	146	HFNAW	39	0.16
143	146	FNAW	40	0.15
144	146	NAW	41	0.25
145	146	AW	42	0.34
146	146	W	43	0.34

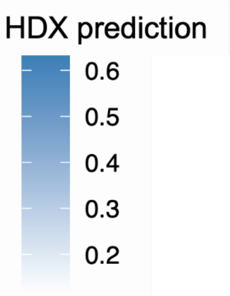

Figure S6. Stepwise fragment predictions of HDX rates in peptide ‘ISVRNSPRTSGTVTVQNHFNAW’ from XYN I. Related to STAR Methods.

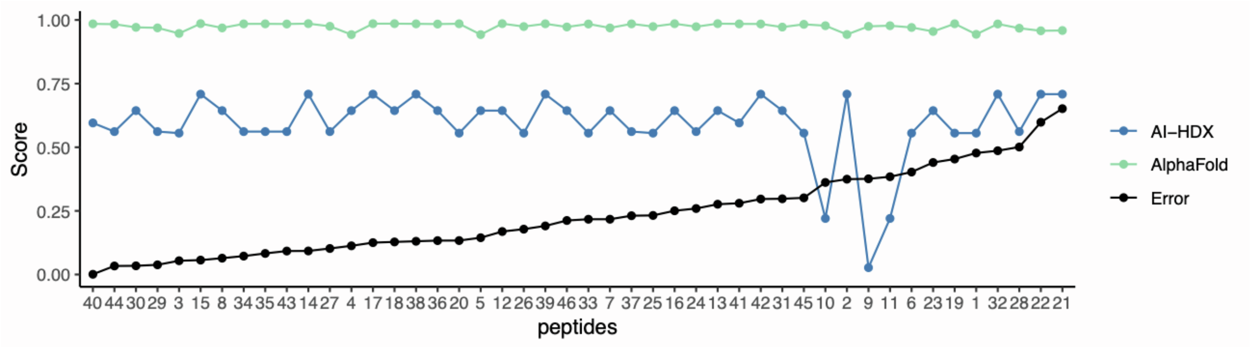

Figure S7. Comparison of AlphaFold2 prediction confidence to AI-HDX prediction confidence. Related to STAR methods.

The confidence index computed from AI-HDX (blue), the confidence score computed from AlphaFold2 (green), and the AI-HDX prediction error compared to experimental HDX rates (black) were plotted.

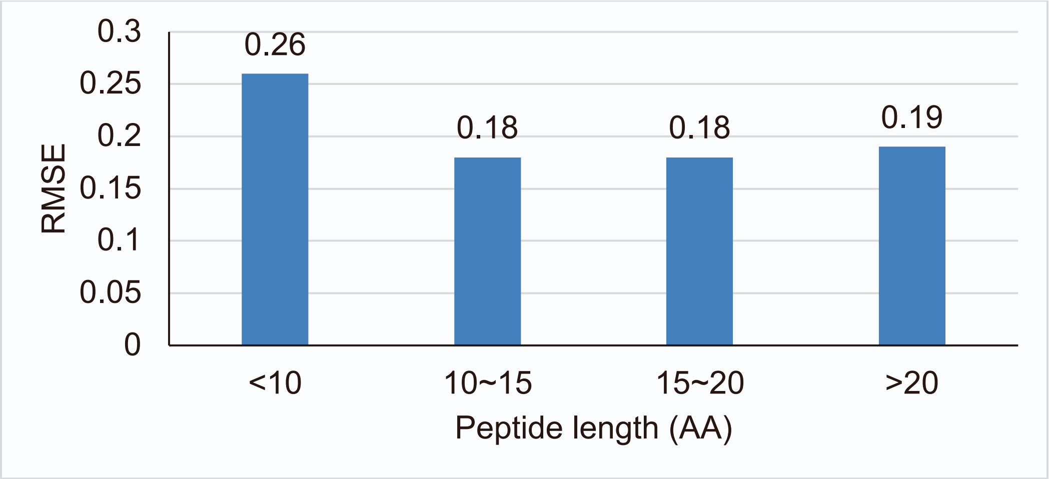

Figure S8. The RMSE of predicted HDX and experimental HDX rates in different peptide lengths in xylanase. Related to STAR Methods and Figure 2.

The peptides with experimental HDX rates in the range of 0.2 to 0.7 were included. The peptides (5 fragments < 10 residues, 12 fragments in 10-15 residues, 11 fragments in 15-20 residues, and 4 fragments > 20 residues) with experimental HDX rates in the range of 0.2 to 0.7 were included.

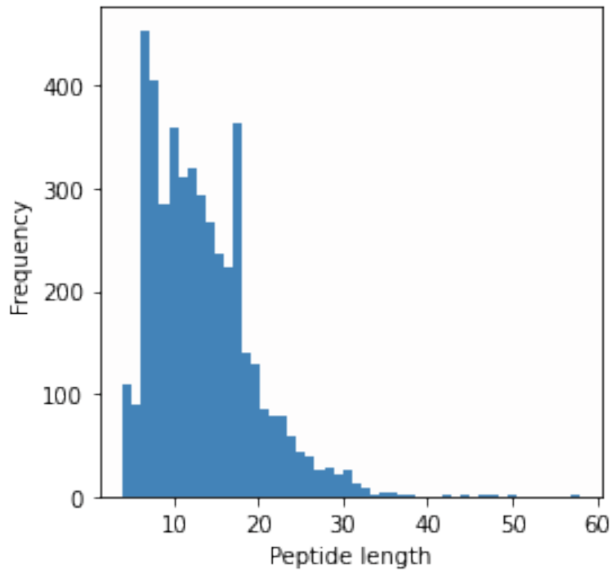

Figure S9. The size distribution of fragment lengths in the curated training dataset. Related to STAR Methods.

Table S1. List of HDX experiments collected and used as the training set for AI-HDX. Related to STAR Methods.

Dataset Identifier	Title	Database	Species	Publication	Lab PI	Announce Date
PXD022172	Characterization of m-lipin using HDX-MS	PRIDE	Mus musculus	[1]	John Burke	6/29/2021
PXD025300	Revealing the dynamic allosteric changes required for formation of the cysteine synthase complex by hydrogen-deuterium exchange mass spectrometry	PRIDE	Escherichia coli	[2]	Kasper Dyrberg Rand	6/7/2021
PXD019884	Glycan-induced structural dynamics in human norovirus P dimers depend on the virus strain and deamidation status	PRIDE	Norovirus Hu/GII.4/MI001/2011/USA, Norovirus Hu/GII/JP/2015/GII.P17_GII.17/Kawasaki308, Norovirus Hu/GII.4/Saga4/2009/JP, Norwalk virus	[3]	Charlotte Uetrecht	4/7/2021
PXD021132	Targeting of disease-linked mutations in PI3K gamma	PRIDE	Homo sapiens	[4]	Dr. John E Burke	3/25/2021
PXD013001	HDX-MS of CSN-CRL2 complexes - Structural basis of Cullin 2 RING E3 ligase regulation by the COP9 signalosome	PRIDE	Homo sapiens	[5]	Argyris Politis	3/4/2021
PXD019530	Molecular determinant for MED1 interaction with the VDR-RXR heterodimer	PRIDE	Homo sapiens	[6]	Sarah Cianferani	2/10/2021
PXD018172	Structural basis for DNA recognition and allosteric control of the Retinoic Acid Receptors RAR-RXR	PRIDE	Mus musculus	[7]	Sarah Cianferani	2/10/2021
PXD019202	Bacillus subtilis RNase M5 cleaves dsRNA in two steps via a two Mg2+-ion dependent mechanism	PRIDE	Geobacillus stearothermophilus	[8]	Sarah CIANFERANI	2/10/2021
PXD019810	HDX-MS of VDR-RXR in complex with ZK168281	PRIDE	Homo sapiens	[9]	Tomas Leek	12/15/2020
PXD020890	HDX-MS of TRAPPII reveals a conserved Rab binding interface	PRIDE	Homo sapiens	[10]	John E Burke	12/10/2020

PXD018007	tRNA binding to KEOPS human protein complex	PRIDE	Homo sapiens	11	David Christopher Schriemer	12/10/2020
PXD017156	The Mechanism of NEDD8 Activation of CUL5 Ubiquitin E3 Ligases	MassIVE	Homo sapiens	12	Elizabeth Komives	12/2/2020
PXD021266	Structure dynamics of ApoA-I amyloidogenic variants in small HDL increase their ability to mediate cholesterol efflux	PRIDE	Homo sapiens	13	Jens O. Lagerstedt	11/20/2020
PXD021743	Dynamics of an LPS translocon induced by substrate and an antimicrobial peptide	PRIDE	Klebsiella pneumoniae	14	Carol V. Robinson	11/16/2020
PXD019047	HDX-MS reveals the perturbed structural dynamics underlie inhibition and altered specificity of the multidrug efflux pump AcrB	PRIDE	Escherichia coli	15	Argyris Politis	10/7/2020
PXD019367	Structural mechanism underlying primary and secondary coupling between GPCRs and the Gi/o family	PRIDE	Homo sapiens	16	Ka Young Chung	7/1/2020
PXD019199	Mapping the CCL8:P672 interface using HDX-MS	PRIDE	Rhipicephalus pulchellus	17	Shoumo Bhattacharya	5/29/2020
PXD018921	HDX-MS analysis of the full human proteasome in complex with regulators	PRIDE	Homo sapiens	18	Julien Marcoux	5/7/2020
PXD018028	c-di-GMP-induced conformational dynamics of the c-di-GMP-binding protein A (CdbA) from Myxococcus xanthus	PRIDE	Myxococcus xanthus (strain DK 1622)	19	Gert Bange	4/16/2020
PXD017095	Stucture and Dynamics of ASB9 CUL-RING Ligase	MassIVE	Homo sapiens	20	Elizabeth Komives	4/15/2020
PXD017575	HDX-MS analysis of Lipin in the presence and absence of anionic membranes	PRIDE	Tetrahymena thermophila SB210	21	John Burke	3/18/2020

PXD013841	Substrate-induced conformational dynamics of the dopamine transporter	PRIDE	Drosophila melanogaster (Fruit fly)	22	Kasper D. Rand	11/11/2019
PXD013594	Investigating the dynamics of the SecA–SecYEG complex dynamic by HDX-MS	PRIDE	Escherichia coli	23	Argyris Politis	7/16/2019
PXD013051	Eukaryotic Ribosome GCN2 interaction Hydrogen/Deuterium Exchange Mass Spectrometry	PRIDE	Oryctolagus cuniculus	24	Roger L. Williams	5/24/2019
PXD010586	Interaction of the GEF SH3BP5 and its cognate GTPase Rab11	PRIDE	Homo sapiens	25	John Edmund Burke	9/18/2018
PXD023434	The box C/D snoRNP assembly factor Bcd1 interacts with the histone chaperone Rtt106 and controls its transcription dependent activity.	PRIDE	Saccharomyces cerevisiae	26	Steve Hessmann	6/1/2021
PXD016391	Structural basis for lipid binding and function by an evolutionally conserved protein, Serum Amyloid A	PRIDE	Mus musculus	27	John R Engen	2/17/2020
PXD011914	Interactions of human norovirus capsid P domain with histo-blood group antigens with Hydrogen-Deuterium Exchange Mass Spectrometry	PRIDE	Norovirus	28	Jasmin Dulfer	3/20/2019
MSV000084200	DECA, a comprehensive, automatic post-processing program for HDX-MS data	MassIVE	Homo sapiens	29	Elizabeth Komives	8/12/2019

Table S2. Protein IDs and protein family information used in this study. Related to STAR Methods and Figure S1.

Uniprot ID	Species	Pfam Accession	Pfam description	Time point used (min)
O60239	Human	PF05276	SH3 domain-binding protein 5 (SH3BP5)	60
P0DI81	Human	PF04628	Sedlin, N-terminal conserved region	60
O43617	Human	PF04051	Transport protein particle (TRAPP) component	60
Q8IUR0	Human	PF04051	Transport protein particle (TRAPP) component	60
P48553	Human	PF12584	Trafficking protein particle complex subunit 10, TRAPPC10	60
P09471	Human	PF00503	G-protein alpha subunit	60
A0A0E4B1P1	Virus	PF00915	Calicivirus coat protein	60
P05388	Human	PF00466	Ribosomal protein L10	50
M5	Bacillus	unknown	NA	60
P61201	Human	PF01399	PCI domain	30
Q9UNS2	Human	PF01399	PCI domain	30
Q92905	Human	PF01398	JAB1/Mov34/MPN/PAD-1 ubiquitin protease	30
Q7L5N1	Human	PF01398	JAB1/Mov34/MPN/PAD-1 ubiquitin protease	30
Q9H9Q2	Human	PF01399	PCI domain	30
Q99627	Human	PF10075	CSN8/PSMD8/EIF3K family	30
Q8N122	Human	PF14538	Raptor N-terminal CASPase like domain	50
Q13098	Human	PF01399	PCI domain	30
P61201	Human	PF01399	PCI domain	30
Q9H9Q2	Human	PF01399	PCI domain	30
Q99627	Human	PF10075	CSN8/PSMD8/EIF3K family	30
Q9P2K8	Human	PF00069	Protein kinase domain	30
P62877	Human	PF12678	RING-H2 zinc finger domain	30
Q06187	Human	PF00779	BTK motif	60
C4T9I0	Klebsiella	PF04453	LPS transport system D	60
Q7K4Y6	Drosophila	PF00209	Sodium:neurotransmitter symporter family	60
Q15369	Human	PF03931	Skp1 family, tetramerisation domain	30
Q04207	Mouse	PF00554	Rel homology DNA-binding domain	60

O95376	Human	PF01485	IBR domain, a half RING-finger domain	60
P54635	Virus	PF00915	Calicivirus coat protein	60
Q9BT78	Human	PF01399	PCI domain	30
P05387	Human	unknown	NA	50
P62873	Human	PF00400	WD domain, G-beta repeat	60
P05386	Human	unknown	DMRT5, DMB domain	50
P0A9D4	Ecoli	PF00132	Bacterial transferase hexapeptide (six repeats)	60
P02647	Human	PF01442	Apolipoprotein A1/A4/E domain	60
Q9Y5R8	Human	PF04099	Sybindin-like family	60
P19793	Human	PF00104	Ligand-binding domain of nuclear hormone receptor	30
P11473	Human	PF00104	Ligand-binding domain of nuclear hormone receptor	30
Q92905	Human	PF01398	JAB1/Mov34/MPN/PAD-1 ubiquitin protease	30
Q7L5N1	Human	PF01398	JAB1/Mov34/MPN/PAD-1 ubiquitin protease	30
L7MC74	Rhipicephalus	unknown	NA	60
P31224	Ecoli	PF00873	AcrB/AcrD/AcrF family	30
Q06187	Human	PF00779	BTK motif	60
P54635	Virus	PF00915	Calicivirus coat protein	60
Q1D489	Myxococcus	unknown	NA	60
Q9UNS2	Human	PF01399	PCI domain	30
Q9BT78	Human	PF01399	PCI domain	30
P35991	Mouse	PF00779	BTK motif	60
P0A1E3	Salmonella	PF00291	Pyridoxal-phosphate dependent enzyme	60
Q9UL33	Human	PF04628	Sedlin, N-terminal conserved region	60
Q9Y296	Human	PF04099	Sybindin-like family	60
Q96Q05	Human	PF08626	Transport protein Trs120 or TRAPPC9, TRAPP II complex subunit	60
Q93034	Human	PF00888	Cullin family	60
P59768	Human	PF00631	GGL domain	60
A0A0J4W1Y0	Klebsiella	PF04390	Lipopolysaccharide-assembly	60
P38772	Yeast	PF04438	HIT zinc finger	60

Q13098	Human	PF01399	PCI domain	30
Q13617	Human	PF00888	Cullin family	30
Q96B36	Human	PF15798	Proline-rich AKT1 substrate 1	50
Q5F4T5	Virus	PF00915	Calicivirus coat protein	60
P80075	Human	PF00048	Small cytokines (intecrine/chemokine), interleukin-8 like	60
Q92731	Human	PF00104	Ligand-binding domain of nuclear hormone receptor	60
P03958	Mouse	PF00962	Adenosine deaminase	60
P36218	T.reesei	PF00457	Glycosyl hydrolases family 11	60

Table S3. Comparison of predicted HDX and experimental HDX rates in SARS-SoV-2 RBD alone and ACE2-bound RBD. Related to Figure 4.

	Predicted rate				Experimental rate *	
peptides	apoRBD	SD	ACE2/RBD	SD	apoRBD	ACE2/RBD
421-431	0.30386943	0.06192482	0.283728182	0.05888822	0.22	0.22
432-449	0.50972545	0.07116771	0.498614937	0.05984097	0.78	0.55
471-486	0.31210095	0.05518444	0.331504315	0.07633545	0.82	0.82
490-510	0.49172077	0.06324586	0.493069559	0.06069119	0.78	0.44

* The exchange rates were calculated from the value of deuterons incorporated in Narang et al., 2021 as:

$$exchange\ rate\ = \frac{Deuterium\ uptake}{theoretical\ deuterium\ uptake\ \times\ dilution\ factor} \div back\ exchange(70\%)$$

$$dilution\ factor\ = 1 - \frac{7.5}{7.5 + 32.5}$$

References

1. Gu, W., Gao, S., Wang, H., Fleming, K.D., Hoffmann, R.M., Yang, J.W., Patel, N.M., Choi, Y.M., Burke, J.E., Reue, K., et al. (2021). The middle lipin domain adopts a membrane-binding dimeric protein fold. Nat. Commun. *12*, 4718.

2. Rosa, B., Dickinson, E.R., Marchetti, M., Campanini, B., Pioselli, B., Bettati, S., and Rand, K.D. (2021). Revealing the Dynamic Allosteric Changes Required for Formation of the Cysteine Synthase Complex by Hydrogen-Deuterium Exchange MS. Mol. Cell. Proteomics *20*, 100098.

3. Dülfer, J., Yan, H., Brodmerkel, M.N., Creutznacher, R., Mallagaray, A., Peters, T., Caleman, C., Marklund, E.G., and Uetrecht, C. (2021). Glycan-Induced Protein Dynamics in Human Norovirus P Dimers Depend on Virus Strain and Deamidation Status. Molecules *26*. 10.3390/molecules26082125.

4. Rathinaswamy, M.K., Gaieb, Z., Fleming, K.D., Borsari, C., Harris, N.J., Moeller, B.E., Wymann, M.P., Amaro, R.E., and Burke, J.E. (2021). Disease-related mutations in PI3Kγ disrupt regulatory C-terminal dynamics and reveal a path to selective inhibitors. Elife *10*. 10.7554/eLife.64691.

5. Faull, S.V., Lau, A.M.C., Martens, C., Ahdash, Z., Hansen, K., Yebenes, H., Schmidt, C., Beuron, F., Cronin, N.B., Morris, E.P., et al. (2019). Structural basis of Cullin 2 RING E3 ligase regulation by the COP9 signalosome. Nat. Commun. *10*, 3814.

6. Belorusova, A.Y., Bourguet, M., Hessmann, S., Chalhoub, S., Kieffer, B., Cianférani, S., and Rochel, N. (2020). Molecular determinants of MED1 interaction with the DNA bound VDR–RXR heterodimer. Nucleic Acids Res. *48*, 11199–11213.

7. Osz, J., McEwen, A.G., Bourguet, M., Przybilla, F., Peluso-Iltis, C., Poussin-Courmontagne, P., Mély, Y., Cianférani, S., Jeffries, C.M., Svergun, D.I., et al. (2020). Structural basis for DNA recognition and allosteric control of the retinoic acid receptors RAR–RXR. Nucleic Acids Res. *48*, 9969–9985.

8. Oerum, S., Catala, M., Bourguet, M., Gilet, L., Barraud, P., Cianférani, S., Condon, C., and Tisné, C. (2021). Structural studies of RNase M5 reveal two-metal-ion supported two-step dsRNA cleavage for 5S rRNA maturation. RNA Biol. *18*, 1996–2006.

9. Rovito, D., Belorusova, A.Y., Chalhoub, S., Rerra, A.-I., Guiot, E., Molin, A., Linglart, A., Rochel, N., Laverny, G., and Metzger, D. (2020). Cytosolic sequestration of the vitamin D receptor as a therapeutic option for vitamin D-induced hypercalcemia. Nat. Commun. *11*, 6249.

10. Jenkins, M.L., Harris, N.J., Dalwadi, U., Fleming, K.D., Ziemianowicz, D.S., Rafiei, A., Martin, E.M., Schriemer, D.C., Yip, C.K., and Burke, J.E. (2020). The substrate specificity of the human TRAPPII complex's Rab-guanine nucleotide exchange factor activity. Commun Biol *3*, 735.

11. Beenstock, J., Ona, S.M., Porat, J., Orlicky, S., Wan, L.C.K., Ceccarelli, D.F., Maisonneuve, P., Szilard, R.K., Yin, Z., Setiaputra, D., et al. (2020). A substrate binding model for the KEOPS tRNA modifying complex. Nat. Commun. *11*, 6233.

12. Lumpkin, R.J., Ahmad, A.S., Blake, R., Condon, C.J., and Komives, E.A. (2021). The Mechanism of NEDD8 Activation of CUL5 Ubiquitin E3 Ligases. Mol. Cell. Proteomics *20*, 100019.

13. Nilsson, O., Lindvall, M., Obici, L., Ekström, S., Lagerstedt, J.O., and Del Giudice, R. (2021). Structure dynamics of ApoA-I amyloidogenic variants in small HDL increase their ability to mediate cholesterol efflux. J. Lipid Res. *62*, 100004.

14. Fiorentino, F., Sauer, J.B., Qiu, X., Corey, R.A., Cassidy, C.K., Mynors-Wallis, B., Mehmood, S., Bolla, J.R., Stansfeld, P.J., and Robinson, C.V. (2021). Dynamics of an LPS translocon induced by substrate and an antimicrobial peptide. Nat. Chem. Biol. *17*, 187–195.

15. Reading, E., Ahdash, Z., Fais, C., Ricci, V., Wang-Kan, X., Grimsey, E., Stone, J., Malloci, G., Lau, A.M., Findlay, H., et al. (2020). Perturbed structural dynamics underlie inhibition and altered efflux of the multidrug resistance pump AcrB. Nat. Commun. *11*, 5565.

16. Kim, H.R., Xu, J., Maeda, S., Duc, N.M., Ahn, D., Du, Y., and Chung, K.Y. (2020). Structural mechanism underlying primary and secondary coupling between GPCRs and the Gi/o family. Nat. Commun. *11*, 3160.

17. Darlot, B., Eaton, J.R.O., Geis-Asteggiante, L., Yakala, G.K., Karuppanan, K., Davies, G., Robinson, C.V., Kawamura, A., and Bhattacharya, S. (2020). Engineered anti-inflammatory peptides inspired by mapping an evasin-chemokine interaction. J. Biol. Chem. *295*, 10926–10939.

18. Lesne, J., Locard-Paulet, M., Parra, J., Zivković, D., Menneteau, T., Bousquet, M.-P., Burlet-Schiltz, O., and Marcoux, J. (2020). Conformational maps of human 20S proteasomes reveal PA28- and immuno-dependent inter-ring crosstalks. Nat. Commun. *11*, 6140.

19. Skotnicka, D., Steinchen, W., Szadkowski, D., Cadby, I.T., Lovering, A.L., Bange, G., and Søgaard-Andersen, L. (2020). CdbA is a DNA-binding protein and c-di-GMP receptor important for nucleoid organization and segregation in Myxococcus xanthus. Nat. Commun. *11*, 1791.

20. Lumpkin, R.J., Baker, R.W., Leschziner, A.E., and Komives, E.A. (2020). Structure and dynamics of the ASB9 CUL-RING E3 Ligase. Nat. Commun. *11*, 2866.

21. Khayyo, V.I., Hoffmann, R.M., Wang, H., Bell, J.A., Burke, J.E., Reue, K., and Airola, M.V. (2020). Crystal structure of a lipin/Pah phosphatidic acid phosphatase. Nat. Commun. *11*, 1309.

22. Nielsen, A.K., Möller, I.R., Wang, Y., Rasmussen, S.G.F., Lindorff-Larsen, K., Rand, K.D., and Loland, C.J. (2019). Substrate-induced conformational dynamics of the dopamine transporter. Nat. Commun. *10*, 2714.

23. Ahdash, Z., Pyle, E., Allen, W.J., Corey, R.A., Collinson, I., and Politis, A. (2019). HDX-MS reveals nucleotide-dependent, anti-correlated opening and closure of SecA and SecY channels of the bacterial translocon. Elife *8*. 10.7554/eLife.47402.

24. Inglis, A.J., Masson, G.R., Shao, S., Perisic, O., McLaughlin, S.H., Hegde, R.S., and Williams, R.L. (2019). Activation of GCN2 by the ribosomal P-stalk. Proc. Natl. Acad. Sci. U. S. A. *116*, 4946–4954.

25. Jenkins, M.L., Margaria, J.P., Stariha, J.T.B., Hoffmann, R.M., McPhail, J.A., Hamelin, D.J., Boulanger, M.J., Hirsch, E., and Burke, J.E. (2018). Structural determinants of Rab11 activation by the guanine nucleotide exchange factor SH3BP5. Nat. Commun. *9*, 3772.

26. Bragantini, B., Charron, C., Bourguet, M., Paul, A., Tiotiu, D., Rothé, B., Marty, H., Terral, G., Hessmann, S., Decourty, L., et al. (2021). The box C/D snoRNP assembly factor Bcd1 interacts with the histone chaperone Rtt106 and controls its transcription dependent activity. Nat. Commun. *12*, 1859.

27. Frame, N.M., Kumanan, M., Wales, T.E., Bandara, A., Fändrich, M., Straub, J.E., Engen, J.R., and Gursky, O. (2020). Structural Basis for Lipid Binding and Function by an Evolutionarily Conserved Protein, Serum Amyloid A. J. Mol. Biol. *432*, 1978–1995.

28. Mallagaray, A., Creutznacher, R., Dülfer, J., Mayer, P.H.O., Grimm, L.L., Orduña, J.M., Trabjerg, E., Stehle, T., Rand, K.D., Blaum, B.S., et al. (2019). A post-translational modification of human Norovirus capsid protein attenuates glycan binding. Nat. Commun. *10*, 1320.

29. Lumpkin, R.J., and Komives, E.A. (2019). DECA, A Comprehensive, Automatic Post-processing Program for HDX-MS Data. Mol. Cell. Proteomics *18*, 2516–2523.

30. Narang, Dominic, D. Andrew James, Matthew T. Balmer, and Derek J. Wilson. 2021. “Protein Footprinting, Conformational Dynamics, and Core Interface-Adjacent Neutralization ‘Hotspots’ in the

SARS-CoV-2 Spike Protein Receptor Binding Domain/human ACE2 Interaction." *Journal of the American Society for Mass Spectrometry* 32 (7): 1593–1600.
